# Developments in the understanding of staging a “major fracture” in polytrauma: results from an initiative by the polytrauma section of ESTES

**DOI:** 10.1007/s00068-023-02245-5

**Published:** 2023-02-23

**Authors:** Yannik Kalbas, Felix Karl-Ludwig Klingebiel, Sascha Halvachizadeh, Yohei Kumabe, Julian Scherer, Michel Teuben, Roman Pfeifer, Hans-Christoph Pape

**Affiliations:** Department of Trauma Surgery and Harald-Tscherne Laboratory, University Hospital Zurich, University of Zurich, Ramistr. 100, 8091 Zurich, Switzerland

**Keywords:** Major fracture, Polytrauma, Treatment strategy, Systematic review, Polytrauma section of ESTES

## Abstract

**Purpose:**

Although the term “major fracture” is commonly used in the management of trauma patients, it is defined insufficiently to date. The *polytrauma section* of ESTES is trying to develop a more standardized use and a definition of the term. In this process, a standardized literature search was undertaken. We test the hypothesis that the understanding of “major fractures” has changed and is modified by a better understanding of patient physiology.

**Methods:**

A systematic literature search of the Medline and EMBASE databases was conducted in March 2022. Original studies that investigated surgical treatment strategies in polytraumatized patients with fractures were included: This included timing, sequence and type of operative treatment. A qualitative synthesis regarding the prevalence of anatomic regions of interest and core factors determining decision-making was performed. Data were stratified by decades.

**Results:**

4278 articles were identified. Of these, 74 were included for qualitative evaluation: 50 articles focused on one anatomic region, 24 investigated the relevance of multiple anatomic regions. Femur fractures were investigated most frequently (62) followed by pelvic (22), spinal (15) and tibial (15) fractures. Only femur (40), pelvic (5) and spinal (5) fractures were investigated in articles with one anatomic region of interest. Before 2010, most articles focused on long bone injuries. After 2010, fractures of pelvis and spine were cited more frequently. Additional determining factors for decision-making were covered in 67 studies. These included chest injuries (42), TBI (26), hemorrhagic shock (25) and other injury-specific factors (23). Articles before 2000 almost exclusively focused on chest injury and TBI, while shock and injury-specific factors (e.g., soft tissues, spinal cord injury, and abdominal trauma) became more relevant after 2000.

**Conclusion:**

Over time, the way “major fractures” influenced surgical treatment strategies has changed notably. While femur fractures have long been the only focus, fixation of pelvic and spinal fractures have become more important over the last decade. In addition to the fracture location, associated conditions and injuries (chest trauma and head injuries) influence surgical decision-making as well. Hemodynamic stability and injury-specific factors (soft tissue injuries) have increased in importance over time.

**Supplementary Information:**

The online version contains supplementary material available at 10.1007/s00068-023-02245-5.

## Introduction

Many advances have been achieved in the care of the polytrauma patient, leading to an improvement in survival and hospitalization rates [1]. In addition, the definition of polytrauma has been refined. Injury severity score (ISS) or related scoring system such as the Hospital Trauma Index (HTI) and later also the New Injury Severity Score (NISS), which are injury-specific classifications, were refined by adding patient specific factors or the physiological responses [2]. In contrast, the term “major fracture” is commonly used but yet to be sufficiently defined [3]. High-energy trauma is commonly associated with extremity fractures and severe tissue lesions. Osseous injuries represent the key focus for the orthopedic surgeon and the timing and technique of fixation is of pivotal importance for patient mobilization [4], pain management [4], the systemic inflammatory response [5] and the overall outcome [4]. Fractures that should be treated with increased priority have long been termed “major fractures” and most frequently, the term had been used for long bone injuries. Recently, the notion to find a universal consensus on the definition of a “major fracture” was introduced [3], motivated by an initiative of the polytrauma section of the European Society for Trauma and Emergency Surgery (ESTES) [6].

In addition, several terminology issues have been addressed in preparing the consensus process, which was initiated at ECTES in 2019, followed by further scientific sessions, in-person discussions and structured discussion groups during courses [3].

A recent survey of this international panel of experienced surgeons suggested that the anatomic location of a fracture should no longer be the only focus of attention. Instead, it has been indicated that a fracture should be considered a “major fracture”, when it drives the surgical treatment strategy [3, 6]. This, in turn, can be brought about by a number of reasons, ranging from physiological derailment [7] to relevant soft tissue injury [8]. These considerations have been supported by numerous examples in the literature and by the latest revision of the abbreviated injury scale: The 2015 version of AIS assigns fractures a higher score in case they are open, or accompanied by a vascular or neurological injury, emphasizing the role of injury-specific factors and the potential for a physiological response [5, 9].

Subsequently, it has been argued that improvements in the terminology may be helpful to develop optimal patient care and Safe Definitive Surgery (SDS) [10, 11]. As part of a consensus process of the polytrauma section of ESTES, we aim to prepare the ground for defining the “major fracture” by revisiting the literature.

This manuscript aims to investigate how fractures have influenced surgical decision-making in the scientific literature. We also intend to identify relevant factors (i.e., physiological, concomitant injuries) that determine treatment strategies and the management of a “major fracture”.

## Methods

The reporting of this systematic review is in accordance with the Preferred Reporting Items for Systematic Reviews and Meta-Analyses (PRISMA) guidelines [12]. We performed a systematic search to identify all relevant original publications that investigated surgical treatment strategy in the polytraumatized patient (operative timing/sequence, type of surgery and decision-making). To approximate how “major fracture” was defined, we assessed the prevalence of the anatomic regions of interest and the core determining factors for the surgical treatment strategy. A qualitative synthesis was performed.

### Search strategy

The search was conducted on March 14, 2022 in the EMBASE and MEDLINE databases: We used a combination of controlled vocabulary and regular search terms. In the Medline Database, we included the Medical Subject Headings (MeSH) “Multiple Trauma” and “fractures, bone” in combination with the terms “fracture”, “polytrauma”, “relevance”, “timing”, “decision” and “major”. In the EMBASE database, the same terms were combined with the EMTREE terms “fracture” and “multiple trauma”. Truncation was used to account for plural forms and alternate spellings. Terms were connected by the Boolean operators and filters were applied to exclude inappropriate article types. In addition to the database search, experts in the field of trauma surgery were asked for potentially relevant studies and references lists of selected studies and related reviews were screened, to identify any important studies missed by the electronic search (Additional sources).

### Extraction and screening

Search results were extracted and documented using EndNote™ version 20 by Clarivate™. Articles were de-duplicated and screened independently by two authors. A cross-check of the extracted data was performed by the senior author. Any disagreement was resolved by a consensus discussion in personal meetings.

### Inclusion criteria

Original studies reported in English or German were assessed for inclusion. Articles were included if they investigated how certain fractures effect the surgical treatment strategy in polytraumatized patients: This concerned timing, sequence or type of surgical interventions and their influence on systemic complications and overall outcome.

### Exclusion criteria

Reviews, letters, commentaries, correspondences, conference abstracts, expert opinions, editorials and in vitro/animal experiments were excluded. Further exclusion criteria were isolated injuries, an ISS < 16, a lack of characterization of study population or injured anatomic regions and a high risk of bias. We also excluded articles, in which the influence of the fracture on the treatment strategy in the polytraumatized patient was insufficiently investigated.

### Retrieval

After initial screening and selection were completed, articles were retrieved from the respective publishers through the access of our universities’ central library. Manuscripts, which could not be retrieved this way, were requested from the central library directly and restored from hard copies when possible. Articles were stored as PDF files in EndNote™ version 20 by Clarivate™.

### Eligibility and risk of bias

Articles were checked for eligibility by three authors and, in case of uncertainty, crosschecked with the senior author in personal meetings. Risk of bias was evaluated and graded as “low”, “moderate” and “high” adapted from the domains of the ROBINS-I tool [13]. Articles with a high risk of bias were excluded.

### Data collection and processing

Data were collected manually and transferred into MS Excel. Besides general metadata, articles were scanned for number of patients, mean injury severity, anatomic regions of interest and whether the focus was on single or on multiple anatomic regions. Further items of interest were other factors determining surgical treatment strategy. Studies were sorted by year of publication. Anatomic regions were stratified into femur, pelvis, acetabulum, spine, tibia and upper extremity (UEX). Number of publications per anatomic region per year were calculated and four time periods of interest were defined: 1982–1989, 1990–1999, 2000–2009 and 2010–2021.

### Data analysis

Data were interpreted qualitatively and represented visually. Fractions and percentages were calculated per time period. Data did not allow for a quantitative synthesis. Statistical analysis and significance testing was not performed.

## Definitions

***Chest injury*** was defined as a relevant injury to the chest that would correspond to an abbreviated injury scale (AIS) or HTI of at least two points. They include, i.e., clinical or radiological lung contusions, serial rib fractures or an initial worsening of respiratory parameters.

***Traumatic brain injury (TBI)*** was defined as a relevant injury to the head that would correspond to an abbreviated injury scale (AIS) or HTI of at least two points.

***Shock*** was defined as hemodynamic instability, measured either through abnormal vital signs (sBP < 90 mmHg, HR > 100), a derailed acid–base balance (lactate > 2 mmol/l, BE < -4) or the need for blood transfusions.

***“Other” relevant injury*** was defined as either the presence of a spinal cord injury (SCI) with a neurological deficit, or the presence of a relevant soft tissue injury including open fractures ≥ type two (Gustilo-Anderson), or the presence of a relevant abdominal injury, either requiring surgical intervention or at high risk for causing hemodynamic instability.

## Results

### Study selection

**Fig. 1:**
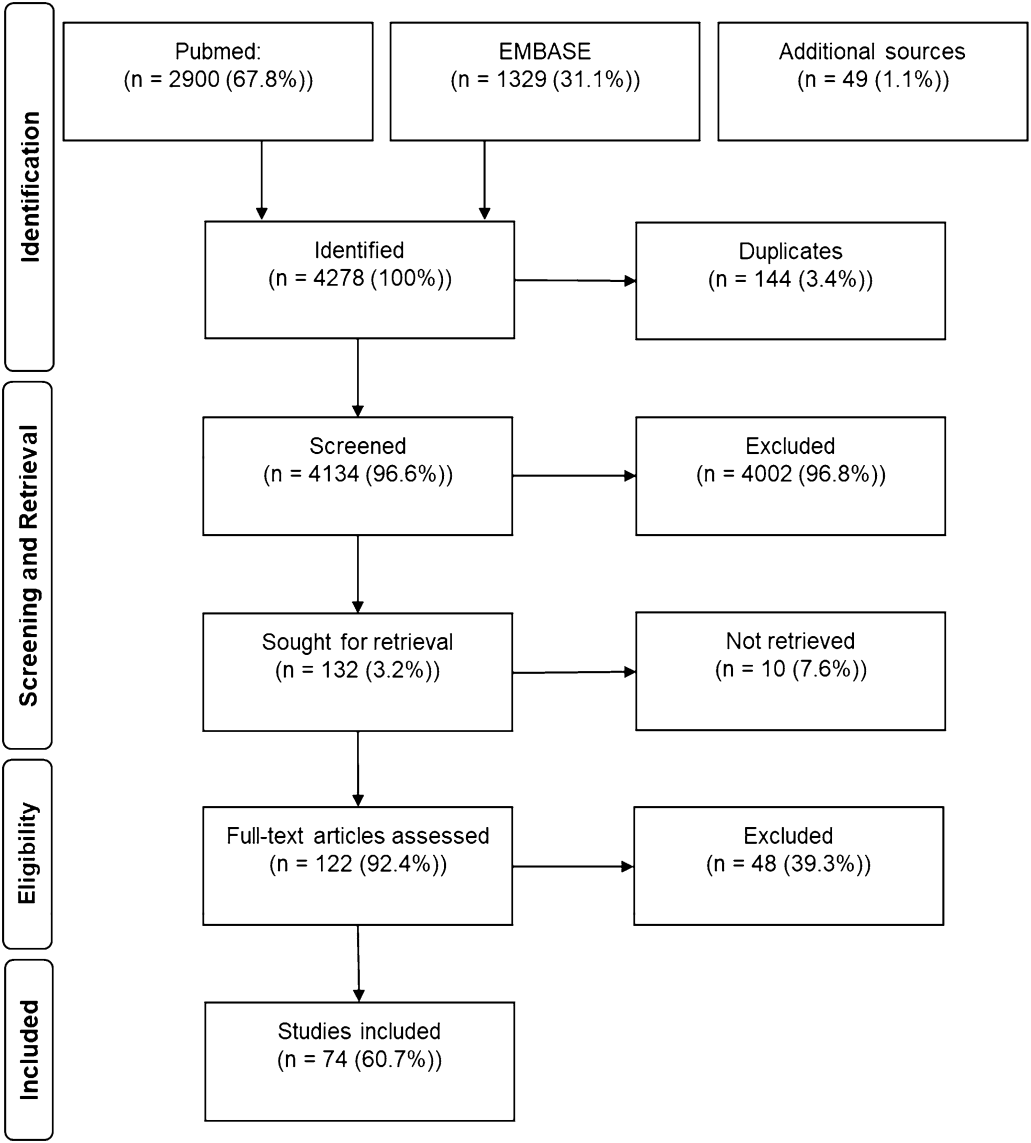
Flowchart of the selection process

The flow chart of the study selection process is presented in Fig. 1: The systematic search in the Medline Database yielded 2900 results and 1329 records were identified through EMBASE. A further 49 records were identified through additional sources. After removal of 144 duplicates, 4134 records were screened and 4002 records were excluded. The remaining 132 articles were sought for retrieval. While 10 articles could not be retrieved, 122 full- text articles were assessed for eligibility. After exclusion of 48 articles, 74 studies remained for qualitative synthesis (complete article information/metadata are presented in the appendix) [4, 7, 14–85].

### Overview

**Fig. 2:**
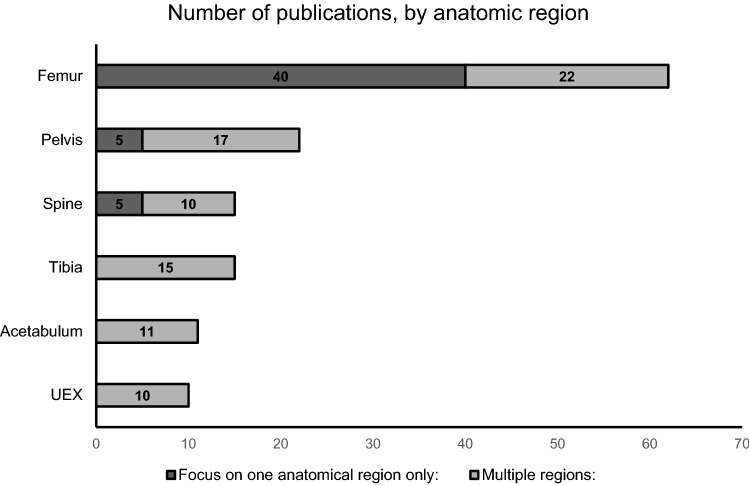
Number of publications per anatomic region, focus on only one vs. multiple regions

A synopsis of all included studies is presented in Table 1. In total, 74 studies published between 1982 and 2022 matched our criteria and were included. The majority were published in the decades from 1990 to 1999 (20 studies) and from 2010 and 2019 (24 studies). Among the included studies, 50 (67.6%) focused on one single anatomic region, while the remaining 24 (32.4%) studies investigated multiple anatomic regions. Among the publications on one anatomic region, 40 (80%) studies focused on femur fractures, 5 (10%) studies on pelvic fractures and 5 (10%) studies on spinal fractures. Among the publications that addressed multiple regions, 22 (91.7%) included femur fractures and 17 (70.8%) included pelvic fractures. Further anatomic regions of interest were tibia, spine, acetabulum and the upper extremities (see Fig. 2 and Table 2).

**Table 1:** Synopsis of general information, relevant anatomic regions and determining factors in all included articles, sorted by year of publication

General information			Relevant anatomic regions							Determining factors			
Authors	Year	*n* =	Focus on	Femur	Pelvis	Acetabulum	Spine	Tibia	UEX	Chest Injury	TBI	Shock	Other
Goris et al. [14]	1982	58	Multiple regions	+				+	+				
Browner et al. [15]	1984	54	One region only	+							+		+
Sturm et al. [16]	1984	207	One region only	+						+			
Johnson et al. [17]	1985	132	Multiple regions	+	+		+			+			
Seibel et al. [18]	1985	56	Multiple regions	+		+				+	+		+
Meek et al. [19]	1986	71	Multiple regions	+				+	+				
Brug et al. [20]	1988	361	One region only	+						+			
Bone et al. [4]	1989	83	One region only	+						+		+	
Burchardi et al. [21]	1990	225	Multiple regions	+				+	+	+			
Nast-Kolb et al. [22]	1990	69	One region only	+						+			
Hofman et al. [23]	1991	58	Multiple regions	+	+		+	+			+		
Pelias et al. [25]	1992	130	Multiple regions	+	+		+			+			
Poole et al. [26]	1992	114	Multiple regions	+				+			+		
Pape et al. [24]	1992	16	One region only	+						+			
Riemer et al. [27]	1992	150	One region only	+									+
Pape et al. [28]	1993	106	One region only	+						+			
Pape et al. [29]	1993	319	One region only	+						+			
Bone et al. [30]	1994	676	Multiple regions	+	+	+		+			+		
Malisano et al. [32]	1994	153	Multiple regions	+				+	+		+		
van Os et al. [33]	1994	27	Multiple regions	+	+	+	+	+	+	+	+		
Charash et al. [31]	1994	138	One region only	+						+			
Reynolds et al. [34]	1995	105	One region only	+						+	+		
Van Der Made et al. [35]	1996	60	One region only	+						+			
Bosse et al. [36]	1997	453	One region only	+						+			
Boulanger et al. [37]	1997	149	One region only	+						+			
Schmidtmann et al. [38]	1997	17	One region only	+						+	+		
Aufmkolk et al. [39]	1998	325	One region only	+						+			
McLain et al. [40]	1999	26	One region only				+						+
Nowotarski et al. [41]	2000	54	One region only	+								+	+
Scalea et al. [42]	2000	327	One region only	+						+	+	+	+
Taeger et al. [45]	2002	45	Multiple regions	+	+			+	+	+	+	+	+
Brundage et al. [43]	2002	674	One region only	+						+	+		
Pape et al. [44]	2002	514	One region only	+						+	+	+	+
Bhandari et al. [46]	2003	1211	Multiple regions	+				+			+		
Nau et al. [47]	2003	352	One region only	+						+	+		
Pape et al. [48]	2003	35	One region only	+						+			
Taeger et al. [50]	2005	409	Multiple regions	+	+			+	+	+	+	+	
Harwood et al. [49]	2005	174	One region only	+						+	+	+	+
Powell et al. [51]	2006	315	One region only	+									+
Pape et al. [52]	2007	165	One region only	+						+	+	+	+
Probst et al. [53]	2007	290	One region only		+								
Morshed et al. [54]	2009	3069	One region only	+									+
O'Toole et al. [55]	2009	227	One region only	+						+			
Tuttle et al. [56]	2009	462	One region only	+							+	+	
Vallier et al. [60]	2010	418	Multiple regions		+	+				+		+	
Hartsock et al. [58]	2010	19	One region only	+						+			
Scannell et al. [59]	2010	205	One region only	+						+			
Enninghorst et al. [57]	2010	45	One region only		+							+	
Schreiber et al. [63]	2011	114	Multiple regions	+	+			+	+				
Pakzad et al. [62]	2011	83	One region only				+						+
Nahm et al. [61]	2011	492	One region only	+						+	+		
Husebye et al. [64]	2012	12	One region only	+									
Vallier et al. [69]	2013	1005	Multiple regions	+	+	+	+			+	+		+
Vallier et al. [70]	2013	1443	Multiple regions	+	+	+	+					+	
Stahel et al. [68]	2013	112	One region only				+						+
Dienstknecht et al. [67]	2013	165	One region only	+						+	+	+	+
Abrassart et al. [65]	2013	70	One region only		+							+	+
Böhme et al. [66]	2013	47	One region only		+							+	
Cantu et al. [71]	2014	2323	One region only	+									
Park et al. [72]	2014	166	One region only				+						+
Steinhausen et al. [73]	2014	379	One region only	+						+		+	
Vallier et al. [76]	2015	335	Multiple regions	+	+	+	+					+	
Konieczny et al. [74]	2015	38	One region only				+			+		+	
Morshed et al. [75]	2015	2949	One region only	+							+		
Reich et al. [77]	2016	376	Multiple regions	+	+	+	+					+	
Glass et al. [79]	2017	294	Multiple regions	+	+	+	+	+	+				+
Byrne et al. [78]	2017	6948	One region only	+									
Pape et al. [7]	2019	3668	Multiple regions	+	+	+	+	+	+	+		+	+
Devaney et al. [80]	2020	1270	Multiple regions		+	+						+	
Tan et al. [85]	2021	103	Multiple regions	+	+			+		+		+	
Denis-Aubrée et al. [81]	2021	201	One region only	+						+	+		+
Feldman et al. [82]	2021	96	One region only	+						+	+	+	+
Höch et al. [84]	2021	989	One region only		+							+	
Flagstad et al. [83]	2021	328	One region only	+						+	+	+	+

+  = Anatomic region/factor in focus

**Table 2:** Number of publications per anatomic regions in respect to focus on one/multiple regions and time periods

	*n* =	Femur	Pelvis	Acetabulum	Spine	Tibia	UEX
**Total**	**74**	**62 (83.7%)**	**22 (29.7%)**	**11 (14.9%)**	**15 (20.3%)**	**15 (20.3%)**	**10 (13.5%)**
Focus on							
One region only	50	40 (80%)	5 (10%)	0	5 (10%)	0	0
Multiple regions	24	22 (91.7%)	17 (70.8%)	11 (45.8%)	10 (41.7%)	15 (62.5%)	10 (41.7%)
Time period							
** 1982–2009**	**44**	**42 (95.5%)**	**8 (18.2%)**	**3 (6.8%)**	**5 (11.4%)**	**11 (25%)**	**7 (15.9%)**
1982–1989	8	8 (100%)	1 (12.5%)	1 (12.5%)	1 (12.5%)	2 (25%)	2 (25%)
1990–1999	20	19 (95%)	4 (20%)	2 (10%)	4 (20%)	6 (30%)	3 (15%)
2000–2009	16	15 (93.8%)	3 (18.8%)	0	0	3 (18.8%)	2 (12.5%)
** 2010–2021**	**30**	**20 (66.7%)**	**14 (46.7%)**	**8 (26.7%)**	**10 (33.3%)**	**4 (13.3%)**	**3 (10%)**

Percentages are calculated within the corresponding strata (i.e., % of the total number “*n*”). Multiple mentions are possible

*UEX* upper extremity

***Femur fractures*** were most frequently addressed and 62 (83.7%) of the 74 studies list it as a relevant fracture—40 studies focused exclusively on the femur. While some studies investigated the role of femoral fractures on the systemic inflammatory response as well as chest or brain injury, other studies compared the effect of immediate versus delayed surgical intervention or compared the effects of reamed and unreamed intramedullary nailing, plate osteosynthesis, and closed reduction external fixation.

***Pelvic fractures*** were the second most common fracture type to be included in this synthesis. Twenty-two (29.7%) studies investigated pelvic fractures in the polytraumatized patient and five studies focused pelvic fractures exclusively. Overall, 14 studies were published after 2010. While some of these studies were concerned with the timing of fracture fixation, other studies focused on the role of instable pelvic ring fractures as the cause for hemodynamic instability.

***Acetabular fractures*** were included in 11 (14.9%) studies, most of them in combination with injuries to the pelvic ring. Only three studies were published before 2010. All subsequent studies were focused on the role of early fracture fixation on the outcome in polytraumatized patients.

***Spinal fractures*** were represented in 15 (20.3%) studies and 5 studies focused on spinal fractures exclusively. The majority of these studies (10 overall, 4 only focus) were published after 2010. Publications dealt with the timing of fracture stabilization in polytraumatized patients and its relevance for the outcome.

***Tibia fractures*** were represented in 15 (20.3%) studies with no studies focusing on tibial fractures only. The vast majority of these studies (11/15) was published before 2010.

***Upper extremity fractures*** were represented the least frequently and addressed in only 10 (13.5%) publications. There were no studies with an isolated focus on the upper extremity. The vast majority of studies (7/10) was published before 2010.

### Determining factors

Besides the fractured anatomic region and general patient characteristics (age, co-morbidities), 67 out of 74 studies focused on at least one core factor, which determined the treatment strategy, or how patients were stratified. These determining factors could be organized into the four upper mentioned categories: Chest injury, TBI, Shock and “other”. An overview is presented in Tables 1 and 3.

**Table 3:** Number of publications per determining factor in respect to focus on one/multiple factors and time periods

	*n* =	Chest injury	TBI	Shock	Other
**Total**	**74**	**42 (56.8%)**	**26 (35.1%)**	**25 (33.8%)**	**23 (31.1%)**
Focus on					
One factor only	39	18 (46.2%)	6 (15.4%)	7 (17.9%)	8 (20.5%)
Multiple factors	28	24 (85.7%)	20 (71.4%)	18 (64.3%)	15 (53.6%)
Time period					
** 1982–1999**	**28**	**19 (67.9%)**	**10 (35.7%)**	**1 (3.6%)**	**4 (14.3%)**
1982–1989	8	5 (62.5%)	2 (25%)	1 (12.5%)	2 (25%)
1990–1999	20	14 (70%)	8 (40%)	0	2 (10%)
** 2000–2021**	**36**	**23 (63.9%)**	**16 (44.4%)**	**24 (66.7%)**	**18 (50%)**
2000–2009	16	10 (62.5%)	9 (56.3%)	8 (50%)	7 (43.8%)
2010–2021	30	13 (43.3%)	7 (23.3%)	16 (53.3%)	11 (36.7%)

Percentages are calculated within the corresponding strata (i.e., % of the total number “*n*”). Multiple mentions are possible

*TBI* traumatic brain injury, *other* spinal cord injury, abdominal injury or open fracture

***Chest injury*** was mentioned as a determining factor in 42 publications and 18 publications focused on chest injury only. Of these 18 publications, 14 were published before 2000. Chest injury remained an important factor after 2000 (23 mentions), though seldom as an isolated entity.

***TBI*** was mentioned in 26 publication and 6 publication focused on TBI as the only determining factor. While the number of mentions increased after 1990, there were no other apparent changes over the years.

***Shock*** was mentioned as a determining factor in 25 publications and 7 publications focused on shock as an isolated entity. Interestingly, there was only one publication mentioning shock before 2000.

***Other*** relevant injuries were included in 23 publications. These factors were mostly injury specific, meaning SCI in connection with spinal fractures and open fractures in connection with long bone fractures. Most publications (18) were published after 2000.

### Changes over time

**Fig. 3:**
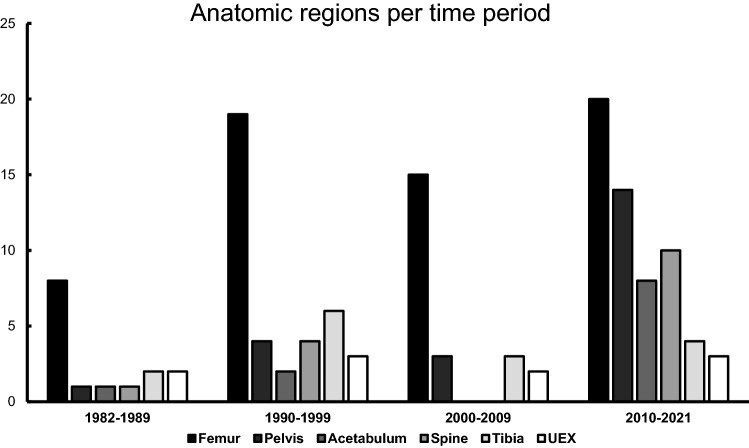
Number of publications per anatomic region sorted by time period

**Fig. 4:**
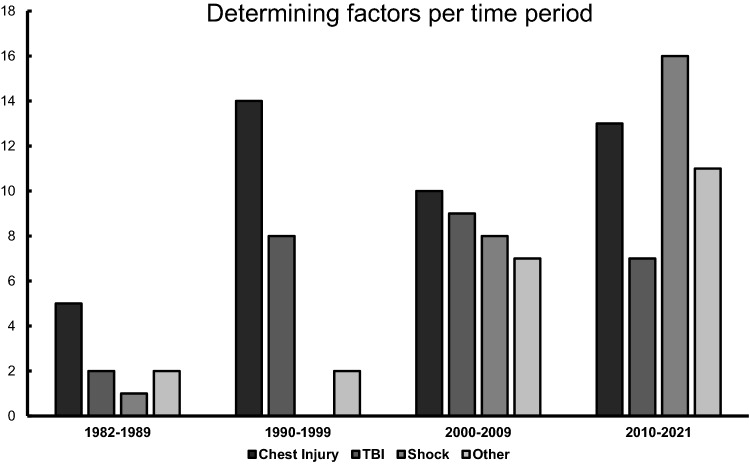
Number of publications on determining factors sorted by time period

We were able to identify relevant changes over time in both the anatomic regions of interest and the determining factors. They are presented in Tables 2 and 3 and Figs. 3 and 4. In regards to determining factors, there has been a remarkable development since the change of the millennia. While TBI and chest injuries remained important determining factors, shock and other relevant injury-specific factors were mentioned a lot more commonly. Furthermore, many publications focused on multiple factors instead of only one. In regards to anatomic regions of interest, the changes occurred mostly since 2010. We noted a steep increase in publications on pelvic, acetabular and spinal fractures, while tibial and UEX fractures decreased. Yet, the femur still remains the most common anatomic region of interest.

## Discussion

In the polytrauma patient, fracture management continues to be of crucial importance, which is represented within the large trauma registries. The German Trauma Registry [TR-DGU], for example, showed that 28.2% of seriously injured patients between 2018 and 2021 suffered severe injury (AIS ≥ 3) to extremities and pelvis, while injuries to the spine were present in 29.6% of cases [86]. The anatomic region of a given injury plays a pivotal role for the surgical treatment strategy. Still, within the last four decades it seems that the focus in the treatment of polytraumatized patients moved beyond considering the injured anatomic region. Instead, surgical treatment strategies are increasingly determined by the local and systemic reaction to trauma and resuscitative efforts as well as individual patient and injury characteristics. Our systematic review demonstrated the following findings:

1. Despite the presence of multiple injuries in the patients addressed in each publication, most of the early publications investigated only a single anatomic region, commonly the femur.
2. Over time, the focus moved away from femur fractures only and pelvic, spine and acetabular fractures are now routinely investigated.
3. In addition to the location of the fracture, in most studies surgical treatment strategy was determined by considering other relevant factors such as chest trauma, TBI or hemodynamic stability.
4. Regarding these determining factors, most authors focused on chest and brain injury before the turn of the century, while afterwards there is an increased focus on hemodynamic stability and other, injury-specific factors.

In terms of the first main finding, femur fractures have long been under special attention due to complication rates associated with their treatment. As delayed fixation and prolonged traction was known to be associated with an increased risk of fat embolism syndrome [18], many early publications dealt with issues of co-factors that may affect the timing of the femoral nailing, especially chest and head injuries [4, 31, 36]. Over time, fractures of the femur were included in all fixation concepts and stabilized in a definitive (intramedullary nailing) or at least temporary (external fixation) fashion. Femur fractures were at the focus of numerous investigations and prospective randomized trials on the merits of damage-control strategies in polytrauma patients [52, 87]. Despite these historical aspects, however, it remains undisputed, that a femur fracture is an important and challenging “major fracture”, and implies high potential for relevant blood loss, systemic inflammatory response and pulmonary complications.

Our second main finding is likely the explained by the improved understanding of the physiology and an increased focus on the role of fracture reduction as a means of surgical resuscitation [84]. This applies especially to the reduction of the pelvic ring to stop infra/retroperitoneal bleeding, as well as realignment of the spinal column and decompression of the spinal cord to treat spinal shock. In case of the pelvis, these might even be performed percutaneously through supraacetabular fixators or sacroiliac screws [8]. As the reduction of the pelvic ring can largely improve the patient’s hemodynamic stability, we feel the importance of pelvic instability may even be underrepresented in the literature. The high potential of pelvic fractures to influence the physiological response can drive surgical treatment strategies and would therefore determine them as “major fractures”.

Another important explanation may be a more patient centered approach and increased focus on favorable functional outcomes through early fixation of pelvic, spinal and acetabular fractures [69]. With the sustained advances in resuscitative and surgical strategies and improvements in training and infrastructure for first responders, many patients can be effectively stabilized despite severe injuries [7, 76, 79]. In such cases, early stabilization of pelvis, acetabulum and spine has shown improved outcomes, thanks to early mobilization and improved patient positioning [69]. This increased focus on the functional outcome has become even more important in case of spinal fractures with spinal cord injuries. Even in borderline patients, these fractures can often be stabilized at highest priority, to establish the best possible neurological/functional outcome [72].

In view of the third and fourth finding, the high importance of the physiological response for surgical decision-making becomes even more apparent. Therefore, it is also very plausible, that many recent publications prioritize injuries to trunk and femur over tibia and the upper extremities. This increased focus on the physiology is also reflected in the grading of an injury (i.e., in the 2015 AIS) [9]. Here, vascular injuries have long been classified independently and are usually scored higher than fractures due to the high potential for a physiological derangement. For fractures, the addition of indicators for potential severe soft tissue injury and/or blood loss might also be helpful. In other scoring systems, such as chest trauma scores, the pure description of osseous injuries, such was serial rib fractures or flail chest was outweighed by adding soft tissue injury (lung contusion) and physiological parameters (i.e., Horovitz ratio) [88]. In the comparison of polytrauma scores, the sole description of anatomic variables appeared to have the weakest predictive value [89]. The addition of physiologic variables led to the Berlin definition of polytrauma, which provides a prediction for patients with a relevant risk of mortality [2]. Further determination of scores demonstrated that the predictive value of an isolated physiologic parameter can be improved by adding further physiologic variables [90]. Therefore, it might also be worth to consider including parameters indicative of patient physiology in the definition of the “major fracture”.

Furthermore, the authors feel that the importance of open fractures, fractures with (neuro-)vascular injuries, fractures with severely contused soft tissues, and amputations have been underrepresented in the literature. It is common practice to address these injuries immediately and with high priority, given the patient’s physiology allows for a damage-control intervention [8].

Finally, one can discuss whether our method used to approximate the definition of “major fractures” in this article is adequate; and there are certainly valuable methods that should be considered in the future to expand on our findings. One important next step to consider would be large registry studies, which could evaluate how different fractures effect the patient’s physiological response.

We feel, however, that the scientific literature gives a good approximation of what is relevant in the clinical field at a given time, which is nicely demonstrated by the increased focus on hemodynamic stability and surgical resuscitation. Therefore, our approach represents an adequate start for further investigations.

## Limitations

We are aware that our study has certain limitations. One of them is certainly the inaccessibility of certain references. This especially concerns some older references, which would have given an important insight into the rationale behind the treatment of polytrauma patients almost 50 years ago. A second important limitation was the large heterogeneity within the included studies in respect to study populations, study designs and outcome parameters. Therefore, categorization with respect to anatomic regions and determining factors was performed by the authors through qualitative interpretation, while an unbiased quantitative synthesis of the data was not possible. A third issue was the limited ability to account for or quantify soft tissue injury, which was partly due to the delayed perception of the importance of soft tissue injuries in the literature but also due to limited documentation.

## Conclusion

Our understanding of the effects of fractures and their operative stabilization on the systemic response and the overall outcome in the polytrauma patients has advanced over the years. While the anatomic regions still plays an important role in determining a “major fracture”, other factors have been included to determine surgical priority. Physiological data, which were underrepresented until the 2000s are now readily available. A weighting between different fractures based on their effects on the physiological response or possible complications, however, has not yet been performed. Furthermore, there is only limited information on the role of soft tissue status or the degree of fracture dislocation or classification. These considerations might prove beneficial in finding a universal consensus. Currently, it appears that all relevant pelvic, spinal and lower extremity fractures should be considered “major fractures” and that the inclusion of the physiological response might be appropriate. The role of soft tissue damage and neurovascular injuries remains to be determined.

### Supplementary Information

Below is the link to the electronic supplementary material.

Supplementary file1 (DOCX 22 KB)

## Data Availability

The datasets generated during and/or analysed during the current study are available from the corresponding author on reasonable request.

## Abbreviations

AIS: Abbreviated injury scale
HTI: Hospital Trauma Index
ISS: Injury Severity Score
NISS: New Injury Severity Score
ESTES: European Society for Trauma and Emergency Surgery
SDS: Safe Definitive Surgery
SCI: Spinal cord injury
TBI: Traumatic brain injury
UEX: Upper extremity

## References

[1] Ruchholtz S (2008). Reduction in mortality of severely injured patients in Germany. Dtsch Arztebl Int.

[2] Pape HC (2014). The definition of polytrauma revisited: an international consensus process and proposal of the new 'Berlin definition'. J Trauma Acute Care Surg.

[3] Kalbas Y, Pape HC (2022). What factors determine a "major fracture"?. Injury.

[4] Bone LB (1989). Early versus delayed stabilization of femoral fractures. A prospective randomized study. J Bone Joint Surg Am.

[5] Pape HC (2022). Pathophysiology in patients with polytrauma. Injury.

[6] Scherer J (2022). Standards of fracture care in polytrauma: results of a Europe-wide survey by the ESTES polytrauma section. Eur J Trauma Emerg Surg.

[7] Pape HC (2019). Timing of major fracture care in polytrauma patients—an update on principles, parameters and strategies for 2020. Injury.

[8] Pfeifer R (2021). Indications and interventions of damage control orthopedic surgeries: an expert opinion survey. Eur J Trauma Emerg Surg.

[9] Association for the Advancement of Automotive Medicine (2018). Abbreviated injury scale: 2015 revision.

[10] Pape HC, Pfeifer R (2015). Safe definitive orthopaedic surgery (SDS): repeated assessment for tapered application of Early Definitive Care and Damage Control?: an inclusive view of recent advances in polytrauma management. Injury.

[11] Pfeifer R (2022). How to clear polytrauma patients for fracture fixation: results of a systematic review of the literature. Injury.

[12] Page MJ (2021). The PRISMA 2020 statement: an updated guideline for reporting systematic reviews. BMJ.

[13] Sterne JA (2016). ROBINS-I: a tool for assessing risk of bias in non-randomised studies of interventions. BMJ.

[14] Goris RJ (1982). Early osteosynthesis and prophylactic mechanical ventilation in the multitrauma patient. J Trauma.

[15] Browner BD, Burgess AR, Robertson RJ (1984). Immediate closed antegrade ender nailing of femoral fractures in polytrauma patients. J Trauma.

[16] Sturm JA (1984). Early osteosynthesis of femoral fractures in multiple trauma: Danger or advantage?. Langenbecks Arch Chir.

[17] Johnson KD, Cadambi A, Seibert GB (1985). Incidence of adult respiratory distress syndrome in patients with multiple musculoskeletal injuries: effect of early operative stabilization of fractures. J Trauma.

[18] Seibel R, LaDuca J, Hassett JM (1985). Blunt multiple trauma (ISS 36), femur traction, and the pulmonary failure-septic state. Ann Surg.

[19] Meek RN, Vivoda EE, Pirani S (1986). Comparison of mortality of patients with multiple injuries according to type of fracture treatment—a retrospective age- and injury-matched series. Injury.

[20] Brug E (1988). Polytrauma and fracture of the femur. Aktuelle Traumatol.

[21] Burchardi H (1990). Organ failure in polytraumatised patients–influence of an early osteosynthesis of fractures on complications. Anasthesie Intensivtherapie Notfallmedizin.

[22] Nast-Kolb D (1990). Surgical management of femoral fractures in multiple trauma. Chirurg.

[23] Hofman PAM, Goris RJA (1991). Timing of osteosynthesis of major fractures in patients with severe brain injury. J Trauma.

[24] Pape HC (1992). Effects of different intramedullary stabilizing procedures of the femur on lung function in polytrauma. Unfallchirurg.

[25] Pelias ME, Townsend MC, Flancbaum L (1992). Long bone fractures predispose to pulmonary dysfunction in blunt chest trauma despite early operative fixation. Surgery.

[26] Poole GV (1992). Lower extremity fracture fixation in head-injured patients. J Trauma.

[27] Riemer BL (1992). Immediate plate fixation of highly comminuted femoral diaphyseal fractures in blunt polytrauma patients. Orthopedics.

[28] Pape HC (1993). Primary intramedullary femur fixation in multiple trauma patients with associated lung contusion—a cause of posttraumatic ARDS?. J Trauma.

[29] Pape HC (1993). Influence of thoracic trauma and primary femoral intramedullary nailing on the incidence of ARDS in multiple trauma patients. Injury.

[30] Bone LB (1994). Mortality in multiple trauma patients with fractures. J Trauma.

[31] Charash WE, Fabian TC, Croce MA (1994). Delayed surgical fixation of femur fractures is a risk factor for pulmonary failure independent of thoracic trauma. J Trauma.

[32] Malisano LP, Stevens D, Hunter GA (1994). The management of long bone fractures in the head-injured polytrauma patient. J Orthop Trauma.

[33] van Os JP (1994). Is early osteosynthesis safe in multiple trauma patients with severe thoracic trauma and pulmonary contusion?. J Trauma.

[34] Reynolds MA (1995). Is the timing of fracture fixation important for the patient with multiple trauma?. Ann Surg.

[35] Van Der Made WJ (1996). Intramedullary femoral osteosynthesis: an additional cause of ARDS in multiply injury patients. Injury.

[36] Bosse MJ (1997). Adult respiratory distress syndrome, pneumonia, and mortality following thoracic injury and a femoral fracture treated either with intramedullary nailing with reaming or with a plate. A comparative study. J Bone Joint Surg Am.

[37] Boulanger BR, Stephen D, Brenneman FD (1997). Thoracic trauma and early intramedullary nailing of femur fractures: Are we doing harm?. J Trauma.

[38] Schmidtmann U (1997). Results of elastic plate osteosynthesis of simple femoral shaft fractures in polytraumatized patients. An alternative procedure. Unfallchirurg.

[39] Aufmkolk M (1998). Influence of primary plate-osteosynthesis of femur fractures on complications in multiple trauma patients with or without thoracic injuries. Unfallchirurg.

[40] McLain RF, Benson DR (1999). Urgent surgical stabilization of spinal fractures in polytrauma patients. Spine (Phila Pa 1976).

[41] Nowotarski PJ (2000). Conversion of external fixation to intramedullary nailing for fractures of the shaft of the femur in multiply injured patients. J Bone Jt Surg.

[42] Scalea TM (2000). External fixation as a bridge to intramedullary nailing for patients with multiple injuries and with femur fractures: damage control orthopedics. J Trauma.

[43] Brundage SI (2002). Timing of femur fracture fixation: effect on outcome in patients with thoracic and head injuries. J Trauma.

[44] Pape HC (2002). Changes in the management of femoral shaft fractures in polytrauma patients: from early total care to damage control orthopedic surgery. J Trauma.

[45] Taeger G (2002). Primary external fixation with consecutive procedural modification in polytrauma. Unfallchirurg.

[46] Bhandari M (2003). Operative management of lower extremity fractures in patients with head injuries. Clin Orthop Relat Res.

[47] Nau T (2003). Fixation of femoral fractures in multiple-injury patients with combined chest and head injuries. ANZ J Surg.

[48] Pape HC (2003). Impact of intramedullary instrumentation versus damage control for femoral fractures on immunoinflammatory parameters: prospective randomized analysis by the EPOFF Study Group. J Trauma.

[49] Harwood PJ (2005). Alterations in the systemic inflammatory response after early total care and damage control procedures for femoral shaft fracture in severely injured patients. J Trauma.

[50] Taeger G (2005). Damage control orthopedics in patients with multiple injuries is effective, time saving, and safe. J Trauma.

[51] Powell JN (2006). Reamed versus unreamed intramedullary nailing of the femur: comparison of the rate of ARDS in multiple injured patients. J Orthop Trauma.

[52] Pape HC (2007). Impact of the method of initial stabilization for femoral shaft fractures in patients with multiple injuries at risk for complications (borderline patients). Ann Surg.

[53] Probst C (2007). Timing and duration of the initial pelvic stabilization after multiple trauma in patients from the German trauma registry: is there an influence on outcome?. J Trauma.

[54] Morshed S (2009). Delayed internal fixation of femoral shaft fracture reduces mortality among patients with multisystem trauma. J Bone Joint Surg Am.

[55] O'Toole RV (2009). Resuscitation before stabilization of femoral fractures limits acute respiratory distress syndrome in patients with multiple traumatic injuries despite low use of damage control orthopedics. J Trauma.

[56] Tuttle MS (2009). Safety and efficacy of damage control external fixation versus early definitive stabilization for femoral shaft fractures in the multiple-injured patient. J Trauma.

[57] Enninghorst N (2010). Acute definitive internal fixation of pelvic ring fractures in polytrauma patients: a feasible option. J Trauma.

[58] Hartsock LA (2010). Randomized prospective clinical trial comparing reamer irrigator aspirator (RIA) to standard reaming (SR) in both minimally injured and multiply injured patients with closed femoral shaft fractures treated with reamed intramedullary nailing (IMN). Injury.

[59] Scannell BP (2010). Skeletal traction versus external fixation in the initial temporization of femoral shaft fractures in severely injured patients. J Trauma.

[60] Vallier HA (2010). Early definitive stabilization of unstable pelvis and acetabulum fractures reduces morbidity. J Trauma.

[61] Nahm NJ (2011). Early appropriate care: definitive stabilization of femoral fractures within 24 h of injury is safe in most patients with multiple injuries. J Trauma.

[62] Pakzad H (2011). Delay in operative stabilization of spine fractures in multitrauma patients without neurologic injuries: effects on outcomes. Can J Surg.

[63] Schreiber VM (2011). The timing of definitive fixation for major fractures in polytrauma–a matched-pair comparison between a US and European level I centres: analysis of current fracture management practice in polytrauma. Injury.

[64] Husebye EE (2012). Intramedullary nailing of femoral shaft fractures in polytraumatized patients a longitudinal, prospective and observational study of the procedure-related impact on cardiopulmonary- and inflammatory responses. Scandinavian J Trauma.

[65] Abrassart S, Stern R, Peter R (2013). Unstable pelvic ring injury with hemodynamic instability: what seems the best procedure choice and sequence in the initial management?. Orthop Traumatol Surg Res.

[66] Böhme J (2013). Polytrauma with pelvic fractures and severe thoracic trauma: does the timing of definitive pelvic fracture stabilization affect the clinical course?. Unfallchirurg.

[67] Dienstknecht T (2013). Do parameters used to clear noncritically injured polytrauma patients for extremity surgery predict complications?. Clin Orthop Relat Res.

[68] Stahel PF (2013). The impact of a standardized "spine damage-control" protocol for unstable thoracic and lumbar spine fractures in severely injured patients: a prospective cohort study. J Trauma Acute Care Surg.

[69] Vallier HA (2013). Do patients with multiple system injury benefit from early fixation of unstable axial fractures? The effects of timing of surgery on initial hospital course. J Orthop Trauma.

[70] Vallier HA (2013). Timing of orthopaedic surgery in multiple trauma patients: development of a protocol for early appropriate care. J Orthop Trauma.

[71] Cantu RV, Graves SC, Spratt KF (2014). In-hospital mortality from femoral shaft fracture depends on the initial delay to fracture fixation and Injury Severity Score: a retrospective cohort study from the NTDB 2002–2006. J Trauma Acute Care Surg.

[72] Park KC (2014). Clinical results of early stabilization of spine fractures in polytrauma patients. J Crit Care.

[73] Steinhausen E (2014). A risk-adapted approach is beneficial in the management of bilateral femoral shaft fractures in multiple trauma patients: an analysis based on the trauma registry of the German Trauma Society. J Trauma Acute Care Surg.

[74] Konieczny MR (2015). Early versus late surgery of thoracic spine fractures in multiple injured patients: is early stabilization always recommendable?. Spine J.

[75] Morshed S, Mikhail C, Miclau T (2015). Timing of femoral shaft fracture fixation affects length of hospital stay in patients with multiple injuries. Open Orthop J.

[76] Vallier HA (2015). Complications are reduced with a protocol to standardize timing of fixation based on response to resuscitation. J Orthop Surg Res.

[77] Reich MS (2016). Is Early Appropriate Care of axial and femoral fractures appropriate in multiply-injured elderly trauma patients?. J Orthop Surg Res.

[78] Byrne JP (2017). Timing of femoral shaft fracture fixation following major trauma: a retrospective cohort study of United States trauma centers. PLoS Med.

[79] Glass NE (2017). Early definitive fracture fixation is safely performed in the presence of an open abdomen in multiply injured patients. J Orthop Trauma.

[80] Devaney GL (2020). Time to definitive fixation of pelvic and acetabular fractures. J Trauma Acute Care Surg.

[81] Denis-Aubrée P (2021). Bilateral femoral shaft fracture in polytrauma patients: Can intramedullary nailing be done on an emergency basis?. Orthop Traumatol.

[82] Feldman G (2021). Evolution of treatment of femoral shaft fracture in polytrauma: Did damage control orthopaedics improve the outcome? A retrospective study. Injury.

[83] Flagstad IR (2021). Factors influencing management of bilateral femur fractures: a multicenter retrospective cohort of early versus delayed definitive Fixation. Injury.

[84] Höch A (2021). Trends and efficacy of external emergency stabilization of pelvic ring fractures: results from the German Pelvic Trauma Registry. Eur J Trauma Emerg Surg.

[85] Tan JH (2021). Definitive surgery is safe in borderline patients who respond to resuscitation. J Orthop Trauma.

[86] TraumaRegister der Deutschen Gesellschaft für Unfallchirurgie. Annual Report 2021

[87] Rixen D (2016). Randomized, controlled, two-arm, interventional, multicenter study on risk-adapted damage control orthopedic surgery of femur shaft fractures in multiple-trauma patients. Trials.

[88] Mommsen P (2012). Comparison of different thoracic trauma scoring systems in regards to prediction of post-traumatic complications and outcome in blunt chest trauma. J Surg Res.

[89] Butcher N, Balogh ZJ (2009). The definition of polytrauma: the need for international consensus. Injury.

[90] Halvachizadeh S (2020). How to detect a polytrauma patient at risk of complications: a validation and database analysis of four published scales. PLoS One.

