# Supplementary material for: Developments in the understanding of staging a “major fracture” in polytrauma: results from an initiative by the polytrauma section of ESTES
Source: Eur J Trauma Emerg Surg. 2023 Feb 23;50(3):657–69. doi: 10.1007/s00068-023-02245-5 (PMC11249440; doi:10.1007/s00068-023-02245-5)
Supplement: Supplementary file 1 — Supplementary file1 (DOCX 22 KB) [file 68_2023_2245_MOESM1_ESM.docx]

***Appendix:***

***Complete information and metadata of the 74 included articles, sorted by year of publication***

| **Ref. Nr.** | **Authors** | **Year** | **Title** | **Journal** | **n =** | **Type of Study** | **ROB** |
| --- | --- | --- | --- | --- | --- | --- | --- |
| 14 | Goris et al. | 1982 | Early osteosynthesis and prophylactic mechanical ventilation in the multitrauma patient | Journal of Trauma | 58 | retrospective | Moderate |
| 15 | Browner et al. | 1984 | Immediate closed antegrade ender nailing of femoral fractures in polytrauma patients | Journal of Trauma | 54 | retrospective | Moderate |
| 16 | Sturm et al. | 1984 | Early osteosynthesis of femoral fractures in multiple trauma: Danger or advantage? | Langenbecks Arch Chir | 207 | retrospective | Moderate |
| 17 | Johnson et al. | 1985 | Incidence of adult respiratory distress syndrome in patients with multiple musculoskeletal injuries: effect of early operative stabilization of fractures | Journal of Trauma | 132 | retrospective | Moderate |
| 18 | Seibel et al. | 1985 | Blunt multiple trauma (ISS 36), femur traction, and the pulmonary failure-septic state | Annals of Surgery | 56 | prospective | Low |
| 19 | Meek et al. | 1986 | Comparison of mortality of patients with multiple injuries according to type of fracture treatment - a retrospective age- and injury matched series | Injury | 71 | retrospective | Moderate |
| 20 | Brug et al. | 1988 | Polytrauma and fracture of the femur | Aktuelle Traumatologie | 361/1003 | retrospective | Moderate |
| 4 | Bone et al. | 1989 | Early versus delayed stabilization of femoral fractures. A prospective randomized study | JBJS | 83/178 | prospective, randomized | Low |
| 21 | Burchardi et al. | 1990 | Organ failure in polytraumatised patients--influence of an early osteosynthesis of fractures on complications | Anästh. Intensivther. Notfallmed. | 225 | retrospective | Moderate |
| 22 | Nast-Kolb et al. | 1990 | Surgical management of femoral fractures in multiple trauma | Chirurg | 69 | prospective | Moderate |
| 23 | Hofman et al. | 1991 | Timing of osteosynthesis of major fractures in patients with severe brain injury | Journal of Trauma | 58 | retrospective | Moderate |
| 25 | Pelias et al. | 1992 | Long bone fractures predispose to pulmonary dysfunction in blunt chest trauma despite early operative fixation | Surgery | 130 | retrospective | Moderate |
| 26 | Poole et al. | 1992 | Lower extremity fracture fixation in head-injured patients | Journal of Trauma | 114 | retrospective | Moderate |
| 24 | Pape et al. | 1992 | [Effects of different intramedullary stabilizing procedures of the femur on lung function in polytrauma] | Unfallchirurg | 16 | prospective | Low |
| 27 | Riemer et al. | 1992 | Immediate plate fixation of highly comminuted femoral diaphyseal fractures in blunt polytrauma patients | Orthopedics | 150 | retrospective | Moderate |
| 28 | Pape et al. | 1993 | Primary intramedullary femur fixation in multiple trauma patients with associated lung contusion - A cause of posttraumatic ARDS? | Journal of Trauma | 106 | retrospective | Moderate |
| 29 | Pape et al. | 1993 | Influence of thoracic trauma and primary femoral intramedullary nailing on the incidence of ARDS in multiple trauma patients | Injury | 319 | retrospective | Moderate |
| 30 | Bone et al. | 1994 | Mortality in multiple trauma patients with fractures | Journal of Trauma | 676 | retrospective | Moderate |
| 32 | Malisano et al. | 1994 | The management of long bone fractures in the head-injured polytrauma patient | JOT | 153 | retrospective | Moderate |
| 33 | van Os et al. | 1994 | Is early osteosynthesis safe in multiple trauma patients with severe thoracic trauma and pulmonary contusion? | Journal of Trauma | 27/57 | retrospective | Moderate |
| 31 | Charash et al. | 1994 | Delayed surgical fixation of femur fractures is a risk factor for pulmonary failure independent of thoracic trauma | Journal of Trauma | 138 | retrospective | Moderate |
| 34 | Reynolds et al. | 1995 | Is the timing of fracture fixation important for the patient with multiple trauma? | Ann Surg | 105/424 | retrospective | Moderate |
| 35 | Van Der Made | 1996 | Intramedullary femoral osteosynthesis: An additional cause of ARDS in multiply injury patients | Injury | 60 | retrospective | Moderate |
| 36 | Bosse et al. | 1997 | Adult respiratory distress syndrome, pneumonia, and mortality following thoracic injury and a femoral fracture treated either with intramedullary nailing with reaming or with a plate. A comparative study | JBJS | 453 | retrospective | Moderate |
| 37 | Boulanger et al. | 1997 | Thoracic trauma and early intramedullary nailing of femur fractures: are we doing harm? | Journal of Trauma | 149 | retrospective | Moderate |
| 38 | Schmidtmann et al. | 1997 | [Results of elastic plate osteosynthesis of simple femoral shaft fractures in polytraumatized patients. An alternative procedure] | Unfallchirurg | 17 | prospective | Moderate |
| 39 | Aufmkolk et al. | 1998 | Influence of primary plate-osteosynthesis of femur fractures on complications in multiple trauma patients with or without thoracic injuries | Unfallchirurg | 325 | retrospective | Moderate |
| 40 | McLain et al. | 1999 | Urgent surgical stabilization of spinal fractures in polytrauma patients | Spine | 26/75 | prospective | Moderate |
| 41 | Nowotarski et al. | 2000 | Conversion of external fixation to intramedullary nailing for fractures of the shaft of the femur in multiply injured patients | JBJS | 54 | retrospective | Moderate |
| 42 | Scalea et al. | 2000 | External fixation as a bridge to intramedullary nailing for patients with multiple injuries and with femur fractures: damage control orthopedics | Journal of Trauma | 327 | retrospective | Moderate |
| 45 | Taeger et al. | 2002 | [Primary external fixation with consecutive procedural modification in polytrauma] | Unfallchirurg | 45/679 | retrospective | Moderate |
| 43 | Brundage et al. | 2002 | Timing of femur fracture fixation: effect on outcome in patients with thoracic and head injuries | Journal of Trauma | 674/1362 | retrospective | Moderate |
| 44 | Pape et al. | 2002 | Changes in the management of femoral shaft fractures in polytrauma patients: from early total care to damage control orthopedic surgery | Journal of Trauma | 514 | retrospective | Moderate |
| 46 | Bhandari et al. | 2003 | Operative management of lower extremity fractures in patients with head injuries | CORR | 1211 | retrospective | Moderate |
| 47 | Nau et al. | 2003 | Fixation of femoral fractures in multiple-injury patients with combined chest and head injuries | ANZ J. Surg. | 352 | retrospective | Moderate |
| 48 | Pape et al. | 2003 | Impact of intramedullary instrumentation versus damage control for femoral fractures on immunoinflammatory parameters: prospective randomized analysis by the EPOFF Study Group | Journal of Trauma | 35 | prospective, randomized | Low |
| 50 | Taeger et al. | 2005 | Damage control orthopedics in patients with multiple injuries is effective, time saving, and safe | Journal of Trauma | 409 | prospective, randomized | Low |
| 49 | Harwood et al. | 2005 | Alterations in the systemic inflammatory response after early total care and damage control procedures for femoral shaft fracture in severely injured patients | Journal of Trauma | 174 | retrospective | Moderate |
| 51 | Powell et al. | 2006 | Reamed versus unreamed intramedullary nailing of the femur: Comparison of the rate of ARDS in multiple injured patients | Journal of Trauma | 315 | prospective, randomized | Low |
| 52 | Pape et al. | 2007 | Impact of the method of initial stabilization for femoral shaft fractures in patients with multiple injuries at risk for complications (borderline patients) | Ann Surg | 165 | prospective, randomized | Low |
| 53 | Probst et al. | 2007 | Timing and duration of the initial pelvic stabilization after multiple trauma in patients from the German trauma registry: is there an influence on outcome? | Journal of Trauma | 290 | retrospective | Moderate |
| 54 | Morshed et al. | 2009 | Delayed internal fixation of femoral shaft fracture reduces mortality among patients with multisystem trauma | JBJS | 3069 | retrospective | Moderate |
| 55 | O'Toole et al. | 2009 | Resuscitation Before Stabilization of Femoral Fractures Limits Acute Respiratory Distress Syndrome in Patients With Multiple Traumatic Injuries Despite Low Use of Damage Control Orthopedics | Journal of Trauma | 227 | prospective | Low |
| 56 | Tuttle et al. | 2009 | Safety and efficacy of damage control external fixation versus early definitive stabilization for femoral shaft fractures in the multiple-injured patient | Journal of Trauma | 462 | retrospective | Moderate |
| 60 | Vallier et al. | 2010 | Early definitive stabilization of unstable pelvis and acetabulum fractures reduces morbidity | Journal of Trauma | 418 | retrospective | Moderate |
| 58 | Hartsock et al. | 2010 | Randomized prospective clinical trial comparing reamer irrigator aspirator (RIA) to standard reaming (SR) in both minimally injured and multiply injured patients with closed femoral shaft fractures treated with reamed intramedullary nailing (IMN) | Injury | 19 | prospective, randomized | Low |
| 59 | Scannell et al. | 2010 | Skeletal traction versus external fixation in the initial temporization of femoral shaft fractures in severely injured patients | Journal of Trauma | 205 | retrospective | Moderate |
| 57 | Enninghorst et al. | 2010 | Acute definitive internal fixation of pelvic ring fractures in polytrauma patients: a feasible option | Journal of Trauma | 45 | retrospective | Moderate |
| 63 | Schreiber et al. | 2011 | The timing of definitive fixation for major fractures in polytrauma--a matched-pair comparison between a US and European level I centres: analysis of current fracture management practice in polytrauma | Injury | 114 | retrospective | Moderate |
| 62 | Pakzad et al. | 2011 | Delay in operative stabilization of spine fractures in multitrauma patients without neurologic injuries: effects on outcomes | Can J. Surg. | 83 | retrospective | Moderate |
| 61 | Nahm et al. | 2011 | Early appropriate care: definitive stabilization of femoral fractures within 24 hours of injury is safe in most patients with multiple injuries | Journal of Trauma | 492/750 | retrospective | Moderate |
| 64 | Husebye et al. | 2012 | Intramedullary nailing of femoral shaft fractures in polytraumatized patients. a longitudinal, prospective and observational study of the procedure-related impact on cardiopulmonary- and inflammatory responses | SJTREM | 12 | retrospective | Moderate |
| 69 | Vallier et al. | 2013 | Do patients with multiple system injury benefit from early fixation of unstable axial fractures? The effects of timing of surgery on initial hospital course | JOT | 1005 | retrospective | Moderate |
| 70 | Vallier et al. | 2013 | Timing of orthopaedic surgery in multiple trauma patients: development of a protocol for early appropriate care | JOT | 1443 | retrospective | Moderate |
| 68 | Stahel et al. | 2013 | The impact of a standardized "spine damage-control" protocol for unstable thoracic and lumbar spine fractures in severely injured patients: a prospective cohort study | Journal of Trauma | 112 | retrospective | Moderate |
| 67 | Dienstknecht et al. | 2013 | Do parameters used to clear noncritically injured polytrauma patients for extremity surgery predict complications? | CORR | 165 | retrospective | Moderate |
| 65 | Abrassart et al. | 2013 | Unstable pelvic ring injury with hemodynamic instability: what seems the best procedure choice and sequence in the initial management? | Orthop Traumatol Surg Res | 70/136 | retrospective | Moderate |
| 66 | Böhme et al. | 2013 | [Polytrauma with pelvic fractures and severe thoracic trauma: does the timing of definitive pelvic fracture stabilization affect the clinical course?] | Unfallchirurg | 47 | retrospective | Moderate |
| 71 | Cantu et al. | 2014 | In-hospital mortality from femoral shaft fracture depends on the initial delay to fracture fixation and Injury Severity Score: a retrospective cohort study from the NTDB 2002-2006 | Journal of Trauma | 2323/7540 | retrospective | Moderate |
| 72 | Park et al. | 2014 | Clinical results of early stabilization of spine fractures in polytrauma patients | Journal of Critical Care | 166 | retrospective | Moderate |
| 73 | Steinhausen et al. | 2014 | A risk-adapted approach is beneficial in the management of bilateral femoral shaft fractures in multiple trauma patients: an analysis based on the trauma registry of the German Trauma Society | Journal of Trauma | 379 | retrospective | Moderate |
| 76 | Vallier et al. | 2015 | Complications are reduced with a protocol to standardize timing of fixation based on response to resuscitation | J Orthop Surg Res | 335 | retrospective | Moderate |
| 74 | Konieczny et al. | 2015 | Early versus late surgery of thoracic spine fractures in multiple injured patients: is early stabilization always recommendable? | Spine | 38 | retrospective | Moderate |
| 75 | Morshed et al. | 2015 | Timing of Femoral Shaft Fracture Fixation Affects Length of Hospital Stay in Patients with Multiple Injuries | Open Orthop J | 2949 | retrospective | Moderate |
| 77 | Reich et al. | 2016 | Is Early Appropriate Care of axial and femoral fractures appropriate in multiply-injured elderly trauma patients? | J Orthop Surg Res | 376 | retrospective | Moderate |
| 79 | Glass et al. | 2017 | Early Definitive Fracture Fixation is Safely Performed in the Presence of an Open Abdomen in Multiply Injured Patients | JOT | 294 | retrospective | Moderate |
| 78 | Byrne et al. | 2017 | Timing of femoral shaft fracture fixation following major trauma: A retrospective cohort study of United States trauma centers | PLoS Med | 6948/17993 | retrospective | Moderate |
| 7 | Pape et al. | 2019 | Timing of major fracture care in polytrauma patients - An update on principles, parameters and strategies for 2020 | Injury | 3686 | retrospective | Moderate |
| 80 | Devaney et al. | 2020 | Time to definitive fixation of pelvic and acetabular fractures | Journal of Trauma | 1270 | retrospective | Moderate |
| 85 | Tan et al. | 2021 | Definitive Surgery Is Safe in Borderline Patients Who Respond to Resuscitation | JOT | 103 | retrospective | Moderate |
| 81 | Denis-Aubrée et al. | 2021 | Bilateral femoral shaft fracture in polytrauma patients: Can intramedullary nailing be done on an emergency basis? | OTSR | 201 | retrospective | Moderate |
| 82 | Feldman et al. | 2021 | Evolution of treatment of femoral shaft fracture in polytrauma: Did damage control orthopaedics improve the outcome? A retrospective study | Injury | 96 | retrospective | Moderate |
| 84 | Höch et al. | 2021 | Trends and efficacy of external emergency stabilization of pelvic ring fractures: results from the German Pelvic Trauma Registry | EJOT | 989 | retrospective | Moderate |
| 83 | Flagstad et al. | 2021 | Factors influencing management of bilateral femur fractures: A multicenter retrospective cohort of early versus delayed definitive Fixation | Injury | 328 | retrospective | Moderate |

**Complete Search Terms:**

**For Medline database via pubmed:**

Search: ("polytrauma*"[Title/Abstract] OR "Multiple Trauma"[MeSH Terms]) AND ("fracture*"[Title/Abstract]) OR ("fractures, bone"[MeSH Terms]) AND ("timing"[Title/Abstract] OR "relevan*"[Title/Abstract] OR "decision*" [Title/Abstract] OR major [Title/Abstract]) Filters: Clinical Study, Clinical Trial, Clinical Trial, Phase I, Clinical Trial, Phase II, Clinical Trial, Phase III, Clinical Trial, Phase IV, Comparative Study, Multicenter Study, Observational Study, Randomized Controlled Trial

**For EMBASE:**

#4 AND ('clinical trial'/de OR 'cohort analysis'/de OR 'comparative study'/de OR 'controlled study'/de OR 'cross sectional study'/de OR 'major clinical study'/de OR 'multicenter study'/de OR 'observational study'/de OR 'outcomes research'/de OR 'prospective study'/de OR 'randomized controlled trial'/de OR 'retrospective study'/de)

#4: #1 AND #2 AND #3

#3: timing* OR 'decision making' OR relevan* OR major

#2: 'fracture'/exp OR 'fracture':ab,ti

#1: 'multiple trauma'/exp OR 'multiple trauma':ab,ti
